# Visibility Graph Based Time Series Analysis

**DOI:** 10.1371/journal.pone.0143015

**Published:** 2015-11-16

**Authors:** Mutua Stephen, Changgui Gu, Huijie Yang

**Affiliations:** 1 Business School, University of Shanghai for Science and Technology, Shanghai 200093, China; 2 Computer Science Department, Masinde Muliro University of Science and Technology, P.O. Box 190-50100, Kakamega, Kenya; Tianjin University, CHINA

## Abstract

Network based time series analysis has made considerable achievements in the recent years. By mapping mono/multivariate time series into networks, one can investigate both it’s microscopic and macroscopic behaviors. However, most proposed approaches lead to the construction of static networks consequently providing limited information on evolutionary behaviors. In the present paper we propose a method called visibility graph based time series analysis, in which series segments are mapped to visibility graphs as being descriptions of the corresponding states and the successively occurring states are linked. This procedure converts a time series to a temporal network and at the same time a network of networks. Findings from empirical records for stock markets in USA (S&P500 and Nasdaq) and artificial series generated by means of fractional Gaussian motions show that the method can provide us rich information benefiting short-term and long-term predictions. Theoretically, we propose a method to investigate time series from the viewpoint of network of networks.

## Introduction

Complex network based time series analysis has attracted noteworthy attention in recent years across various domains. By mapping a time series to a network, one can investigate visually the structural patterns at different time scales from microscopic to macroscopic levels [1]. As a result, several novel ways have been proposed on how to convert time series data into complex networks. Zhang et al. [2–4] construct a network from pseudo periodic time series where each cycle is represented by a single node, and a threshold is set to link node pairs with strong cross-correlations. From the viewpoint of phase space reconstruction, one can also take all the possible series segments with a certain length as nodes. Xu et al propose an approach that links each node with its closest *k* neighbors [5–8]. While in the references [9, 10] the nodes are networked according to the correlation strength between the nodes, which turns out to be a special case of the widely used recurrence network approach [11–24]. Lacasa *et al* propose the widely used visibility graph algorithms [25–35] by linking visible elements in a series. Each of these approaches was quickly adopted and widely used among various researchers to extract information embedded in time series from varied domains.

However, in the cited methods, a time series is projected to a static network. As a result, one can hardly find the evolutionary behaviors of the system. A complicated system contains generally many elements, monitoring which produces a multivariate time series. In literature, several novel methods are designed to extract from segments of the multivariate time series relationship networks between the elements, as being the state representatives of the corresponding time intervals. To cite examples, Munnix et al. [36] use the correlation matrix between stocks to represent state of a stock market; In reference [37] Zheng, et al. employ the principal component analysis to extract further believable information from the cross-correlation matrix; Gao et al. [16, 17, 20, 21] embed a multivariate time series in a multi-dimensional phase space, and calculate correlation between each pair of the phase tensors. Each segment of series corresponding to each variate is mapped to a node. Strong relationships are reserved by introducing different thresholds to filter out links between segments coming from the same series and from different series, respectively. By this way, the recurrence network approach is extended to investigate multivariate series; Buccheri et al. [38] construct from the correlation matrix the plenary maximally filtered graphs (an extension of the simple spanning-tree), in which the largest weights are retained while constraining the subgraph to be globally a planar graph; While Gao [39, 40] conduct linear regressions of every element to other elements and the set of values of fitting parameters are used to measure local states. The successive occurring states are then linked into a transmission network.

The motivation of the present work is twofold. First, we try to find evolutionary behaviors of a system embedded in one-dimensional time series. The existing algorithms to map a time series to a network are generally designed in the framework of phase space reconstruction. For a deterministic dynamical system, one can determine the embedding dimension of the system *m*. All the segments with length *m* in the original time series are depicted in the phase space, as being the states visited in the dynamical process. Every pair of states is linked if they are close enough, which results into a network (e.g., recurrence network). But in the downstream procedures of constructing networks, detailed information stored in the states is used in a rough way. For example, in the recurrence plots one calculate the Euclidean distance between each pair of the segments to measure the relationship between the states, in which the state information is all lost except the distance. This roughness in using the information embedded in the states covers up some interesting dynamical behaviors and this leads the focus only to the global characteristics. Consequently, the states are not properly distinguished hence one cannot monitor the system’s evolutionary behavior.

Quite often in practice, people are interested in the short-term prediction of a state. For instance, one maybe interested in the present state of a stock, and its probable state in the following week. He may be concerned about the intensity of increase or decrease with reference to historical data, an investigation that may not be obviously attained from literature review. This guides our second motivation in this work in lieu of providing as concise as possible information from a series segment (state).

In order to address the above stated motivations, we endeavor to extract state patterns concisely while preserving the internal characteristics, and explore the transfer relations between the distinguishable states. Contributions of the present work thus include,

We propose a method to construct a transmission network from a one-dimensional time series, in which the nodes are the local states and the links the transfers between states. Technically, from an initial time series, one can extract all the segments in the series with a predefined window size. We map each segment to a visibility graph, which is used to represent the state of the system at the corresponding time interval. The successively occurring visibility graphs are linked in turn, which leads to a transfer network of distinguishable states. The weights of links reflect the transfer behaviors of the distinguishable states. This algorithm thus produces a ‘network of visibility graphs’.

Some interesting findings are found from empirical data. To illustrate the functionality of our algorithm, we investigate the Nasdaq and S&P500 daily stock indices. We find several motifs, i.e., the states occurring with significant high frequencies compared with that in the shuffled series. Similarly, there are several hub nodes, which occur with significantly high frequencies compared with that of the other nodes. A transfer loop is also found between the hubs, which can be used in short-term prediction, i.e, from the state at present time one predicts what will happen at the next time step. Some motifs are positioned in the time series according to fractal behaviors, exhibiting long-term persistence of the time series. To validate our approach, we also conduct detailed calculations for artificial time series generated with fractional Gaussian motions, as being reference to understand the results for empirical data. The fractional Gaussian motions exhibit similar characteristic behavior with that in recordings of the stock markets.

In a nutshell, in the present work we propose a approach to convert a time series to a temporal network as well as a network of visibility graphs. By this way, the theories and tools in the two speedy developing branches of complex network (network of networks [41] and temporal networks [42]) can be extended to the field of time series analysis.

## Method and Materials

### Represent local States with visibility graphs

Let a window with size *s* slide along a time series, {*y*_1_, *y*_2_, …, *y*_*N*_}. The covered segments read as follows;

$$\begin{array}{ccc} Y_{k} & = & (y_{k},y_{k+1},\cdots ,y_{k+s-1}),\\ k & = & 1,2,\cdots ,N-s+1. \end{array}$$ (1)

We propose the visibility graph as the tool to extract structural information embedded in the segments. The resulting visibility graphs can capture the key structural characteristics of the corresponding segments, and consequently are taken as the description of the local sates in different time durations.

Now we map the segment *Y*_*k*_ to a visibility graph [25]. Each data value is considered to be a node. Two nodes are connected if they can see each other, namely, a straight visibility line exists between them. Formally, two arbitrary data values *y*_*a*_ and *y*_*b*_ are visible to each other if each point *y*_*c*_ between them satisfies the criterion;

$$\begin{array}{c} y_{c}\leq y_{b}+\left(y_{a}-y_{b}\right).\frac{b-c}{b-a}. \end{array}$$ (2)

The constructed visibility graph can be represented with an adjacency matrix, *g*_*k*_, whose element *g*_*k*_(*a* − *k* + 1, *b* − *k* + 1) equals 1(0) if *y*_*a*_ and *y*_*b*_ are visible (invisible). Here, the identification numbers of the nodes corresponding to *y*_*a*_ and *y*_*b*_ are assigned to be *a* − *k* + 1 and *b* − *k* + 1, to be sure they are in the interval of [1, *s*]. This results into an *s* by *s* matrix for the *k*th segment. Covering the whole series, a set of adjacency matrices, *G* = {*g*_1_, *g*_2_, …, *g*_*N*−*s*+1_} is obtained.

### State transfer network

Here, we define a state transfer network to describe transfer probabilities between distinguishable local states. In the time series, if a state at time *b* occurs immediately after another state at time *a*, then we construct a directional link from *g*_*a*_ to *g*_*b*_. Accordingly, the link means a transfer from one state to the other state. By using this procedure a state chain with directional links is attained, which reads;

$$\begin{array}{c} g_{1}\to g_{2}\to \ldots \to g_{N-s+1} \end{array}$$ (3)

Here we find out all the distinguishable states. Let us scan through *G* comparing each state with the others. If any two states are identical (their adjacency matrices are the same) one replaces the later one with the the reference state. For instance, if *g*_1_ = *g*_4_, the state *g*_4_ is replaced with *g*_1_. This process is done iteratively for all states. The survival states are unique states, which are defined to be nodes. We reckon the number of links between each pair of the nodes (survival states), which is the weight of the link between them. By this procedure, the time series is mapped further to a network of distinguishable states (visibility graphs), called state transfer network, with edge direction being the transfer direction and edge weight being the transfer times.

### Properties of the state transfer network

Herein, we are interested in several properties of the state transfer network, including,

*Occurring frequency*. The occurring frequency of a node in the duration of recording is herein called *degree*. A hub node means its occurrence number is significantly larger compared with that of the other nodes. Though hubs are clearly observed, the behavior may be common even in null models hence holding less non-trivial characteristics. If the occurring frequency of a node in the original time series is significantly larger than that in a shuffled time series, the node is called *motif* [43], which can be used as a global representative of the time series.

*Transmission probability*. Strong correlation usually exists in time series, which means occurrence of a state depends strongly on the previous states rather than its occurring stochastically. We expect there exist significant large link weights between some hubs or motifs, which can be greatly helpful in short-term prediction, i.e, based upon the state at present time one predicts what will happen at the next time step.

*Long-term persistence*. A large amount of research works have reported the self-similar structures of time series in diverse research fields (see, e.g., [44]). This kind of fractal behavior makes it possible for us to predict behaviors of complex systems in macroscopic time scales. We will show that the fractal structure can be displayed by the occurring positions of some motifs on time series.

The re-scaled range analysis (R/S) is used [45]. For a specified motif, one can record the positions it occurs at, denoted with *ω*_*k*_, *k* = 1, 2, ⋯, *M*, where *M* is the occurring frequency of the motif. The increment series reads, *ω*_*k*+1_ − *ω*_*k*_, *k* = 1, 2, ⋯, *M* − 1. All the possible segments with length *n* read, *Ω* ^*j*^ ≡ (*ω*_*j*+1_ − *ω*_*j*_, *ω*_*j*+2_ − *ω*_*j*+1_, ⋯, *ω*_*j*+*n*_ − *ω*_*j*+*n*−1_), *j* = 1, 2, ⋯, *M* − *n*. The corresponding accumulated departures for the *j*th segment can be constructed,

$$\begin{array}{ccc} & & \Phi^{j}\left(i\right)\equiv \sum_{w=1}^{i}\left[\Omega^{j}\left(w\right)-<\Omega^{j}>\right]=\omega_{j+i}-\omega_{j}-\frac{i}{n}\left(\omega_{j+n}-\omega_{j}\right),\\ & & \;i=1,2,\cdots ,n. \end{array}$$ (4)

The re-scaled range is estimated as,

$$\begin{array}{ccc} R/S(n) & \equiv & <R^{j}/S^{j}\left(n\right)>\\ & = & \frac{1}{M-n}\sum_{j=1}^{M-n}\frac{max\left[\Phi^{j}\left(1\right),\Phi^{j}\left(2\right),\cdots ,\Phi^{j}\left(n\right)\right]-min\left[\Phi^{j}\left(1\right),\Phi^{j}\left(2\right),\cdots ,\Phi^{j}\left(n\right)\right]}{std(\Omega^{j})}. \end{array}$$ (5)

If there exists scaling invariance in the occurring position series, we have *R*/*S*(*n*)∼*n* ^*δ*^, where *δ* is the Hurst exponent.

### Why use visibility graph to measure local states?

It should be pointed out that using visibility-graph as being state representative is not a trivial selection. Obviously, rather than the visibility-graph we have alternative methods to extract the state information. For example, one can simply compare the values of successive elements in a segment and record the increasing, keeping unchanged, and decreasing with +1, 0, and −1, respectively. By this way each segment is symbolized to a series of discrete values.

The advantages of the visibility-graph include, (1) It can capture precise information of a state. Comparing with the symbolizing procedure, the visibility-graph can extract detailed information of sub-segments at different scales in each segment, at the same time keeps reasonably simple. On the contrary, the symbolizing procedure can only reserve the immediate increase/decrease information; (2) It can be used in analyzing non-stationary time series. A stochastic process, e.g., the fractional Brownian motion, is generally non-stationary, which makes the probability distribution function of the phase vector (series segment) time-dependent [22]. Accordingly, if we use an improper solution to represent states (e.g., the original phase vector in multi-dimensional phase space), the estimations of transfer probabilities (the links) between the states may change with time. Fortunately, this non-stationary effect is eliminated effectively by using visibility graph. Because a segment is very short, its trend can be mainly described with a straight line. Accordingly, visibility graph for the corresponding de-trended segment is identical with that for the original segment, called invariance under affine transformations [25]).

### Window size selection

How to select a proper value of the window size, *s*, is a key problem in the present algorithm. If it is unreasonably small, the number of state patterns is too limited to distinguish different states. On the other hand, the number of state patterns will increase geometrically with the increase of the window size. A larger set of state patterns requires subsequently a longer time series to guarantee statistical significance of the transfer properties, motifs, hubs, and long-term persistence. However, even so, the computational time increases requiring high performance computers to execute, which may not always be readily available.

For deterministic dynamics, a reasonable way is to use the embedding dimension of the considered time series determined in the framework of phase space reconstruction. But for stochastic processes such as the fractional Brownian motion, one meets an essential problem, i.e, the embedding dimension is infinite [22]. Any finite embedding dimension leads to loss of relevant information. Therefore, phase-space reconstruction can not provide us a unified framework to determine the value of the window size *s*.

Stimulated by the interpretation of the recurrence network’s approach to stochastic processes [22], here we propose to understand our approach in an alternative way analogous with the ideas in random geometric network [46]. For a time series, no matter whether it is generated by a deterministic or a stochastic process, we specify an embedding dimension *s*, and embed the series in the *s*-dimensional phase space. Properties of a state transfer network could be computed solely from the occurrent sequence of visibility graphs corresponding to the phase vectors (segments) of the dynamical process. The deviations of a special case from the expectations come from the statistical dependencies between the different embedding components, the finite samples, and the finite-scale effects. Comparing the results for the original series with that for the shuffled and/or theoretical modelling series will show us nontrivial properties of the investigated process.

Hence, the criterions for the selection of the window size include, (1) It is large enough to distinguish different states; (2) It is small enough to be sure the state transfer network and the subsequent structural characteristics are statistically significant; (3) For deterministic dynamical processes the minimum embedding dimension can be used as a reference; (4) If there exists a natural period (or people are interested in a special period), this period should be selected as the window size. The key point is that a proper window size can help us find non-trivial characteristics embedded in the original series comparing with that in the shuffled/modelling series.

### Data

We investigate fractional Brownian motions [47, 48] as being the reference to understand the results for series in reality. A fBm refers to a continuous-time Gaussian process whose characteristics depend on its Hurst exponent 0 ≤ *H* ≤ 1. It is scale-invariant, namely, the probability distribution function of its increment *x*(*t* − *s*) ≡ *fBm*(*t*) − *fBm*(*s*) satisfies $∼\frac{1}{{\left|t-s\right|}^{H}}F(\frac{x}{{\left|t-s\right|}^{H}})$. It has a convergent variance of increment that obeys a power-law, *Var*[*x*(*t* − *s*)] ∼ |*t* − *s*|^2*H*^. The built-in program *wfbm*.*m* in *Matlab* is used to generate the fBm series. For each generated fBm series, we consider in calculations the increment series, called fractional Gaussian motion (fGm). The increment series is stationary.

Obtained from yahoo finance [49], we use the daily closing value of Nasdaq and S&P500 stock indices ranging between 3^rd^ January, 2000 and 14^th^ November, 2014. Each contains a set of 3742 original data records. Daily return series is considered, i.e.,

$$\begin{array}{ccc} Y_{k} & = & log\left(p_{k+1}\right)-log\left(p_{k}\right),\\ k & = & 1,2,\cdots ,3741, \end{array}$$ (6)

where *p*_*k*_ is the value of stock index.

The length of segment, *s*, is selected to be 5 for the generated fGm series and the stock market series. For the stock markets, there exists a natural period of 5 days (one week). For the fractional Gaussian motions the reason is three-fold. First, the fractional Brownian motion is widely used to mimic stock price in mathematical finance, such as that in the fractional Black&Scholes pricing model (see, e.g., [50]). Window size is selected to be 5 to provide a comparable reference to understand the results for the stock markets. Second, this selection leads to a total of 25 state patterns. For a series with about four thousand length, the average occurring number for each pattern is about 160. Deviations of real occurring numbers of the patterns from the average value are statistically significant. Third, by using two different methods for determining embedding dimension [51, 52], the numerical results suggested that the minimum embedding dimension for fGm series with several thousands length is 5 [22, 53], though it is pointed out that this result is length-dependent and may lead to serious tricks in the framework of phase-space reconstruction. Here it is used just as a reference.

For comparison purpose we present also the calculation results for *s* = 6. As for the selection of *s* = 4, there appear only four visibility graphs, so limited number of which can not distinguish the two stock markets (not shown).

## Results

As stated above, the window size, s = 5, was deemed appropriate to unearth the nontrivial characteristics buried within the original series. Herein, we provide the results for both reference and empirical data using the preferred window size; but also include results for window size, s = 6 for comparison purposes.

### Window size *s* = 5

**Fig 1:**
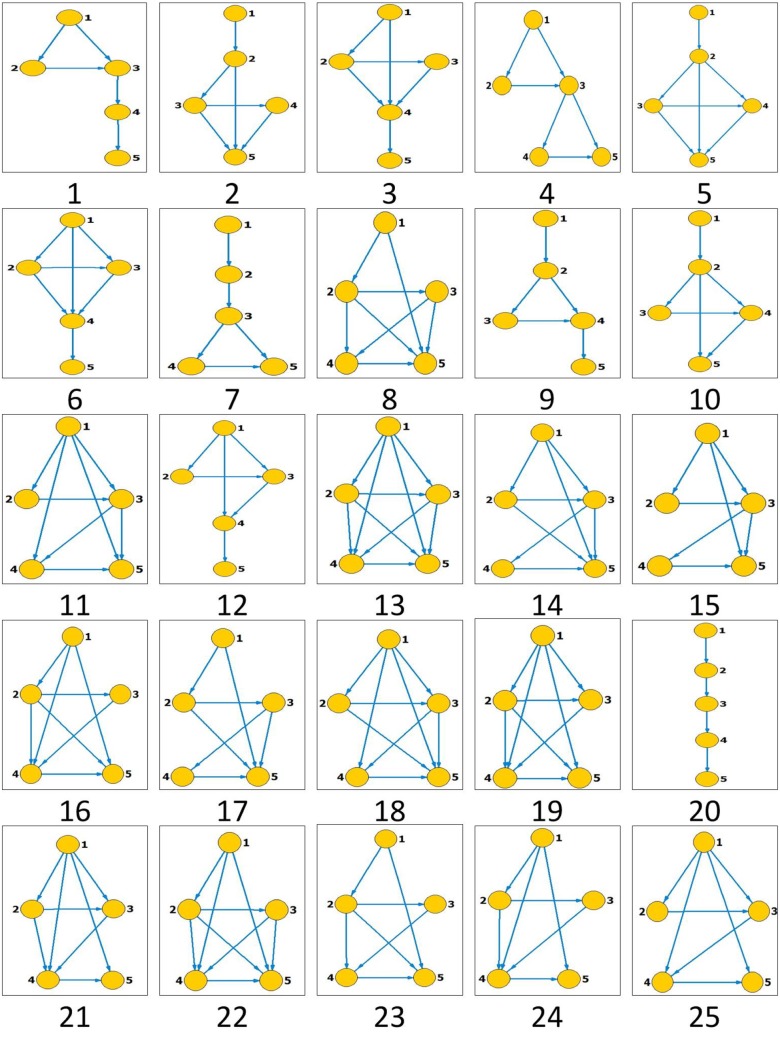
All the states occurring in the state transfer networks for the fGm and stock market index series. Segment length is selected to be *s* = 5. Each state is assigned an identifier number as presented below it.

Fig 1 illustrates the total of 25 local states (visibility graphs) that occur in the considered fGm and stock market series, where each is assigned a unique identifier number.

#### Fractional Gaussian motions

**Fig 2:**
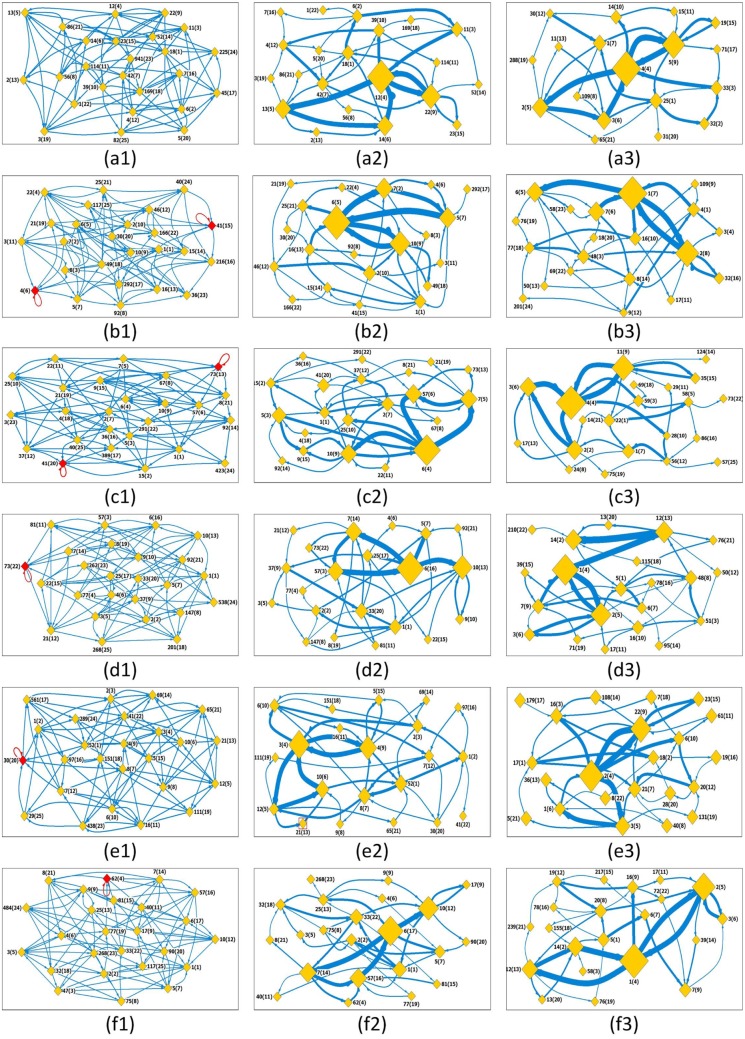
State transfer networks for fGms series. Segment length is selected to be *s* = 5. Subplots (a1)-(f1) are the original state transfer networks for the fGm series with *H* = 0.5, 0.6, 0.65, 0.7, 0.75, and 0.8, respectively. The nodes that have self-links are marked with red color. The label *x*(*y*) means the state occurs for the first time at the position *x* along the time series (the *x*’th segment), and its identifier number is *y*; (a2)-(f2) The strong state transfer networks constructed by filtering out weak links (less than 25) in the original state transfer networks; (a3)-(f3) Shuffled networks. One can shuffle each original fGm series, and construct from the resulting series a shuffled network. Each displayed shuffled network is an average over 1000 realizations. Weak links also are filtered out. Except in the networks shown in (a1)-(f1), the size of a node indicates the occurring degree of the state. The width of an edge is the link’s weight.

The first column in Fig 2, i.e., the subplots of Fig 2(a1)–2(f1), show the state transfer networks for fGm series with Hurst exponents *H* = 0.5, 0.6, 0.65, 0.7, 0.75 and *H* = 0.8, respectively. The length of the generated series is 4000 (to be comparable with that for the stock index series). For visual convenience the weak links (whose strength ≤25) are filtered out, which results into the so-called strong networks, as shown in the subplots of Fig 2(a2)–2(f2). For each original series, one can shuffle it and reconstruct a state transfer network for the shuffled series. State transfer networks averaged over 1000 shuffling realizations each are presented in the third column in Fig 2, including the subplots of Fig 2(a3)–2(f3) respectively, called shuffled networks. In the strong and shuffled networks, the size of a node indicates the occurring degree of the visibility graph and the width of a link indicates the edge’s weight. The label *x*(*y*) means the visibility graph numbered *y* occurs for the first time at position *x* along the time series.

For *H* = 0.5, in the strong network one can find four hubs with Nos 12(4), 22(9), 13(5) and 14(6), which are linked by significant strong edges into a pattern. A simple comparison show that this pattern reoccurs almost exactly in the shuffled network. Actually, except some structure-details formed by weak edges, the pattern of the shuffled network is identical with that for the strong network, i.e., the structural behaviors of the strong network are series-element-order independent. We will not consider the case of *H* = 0.5 for further calculations. Herein, we take the results for *H* = 0.65 and *H* = 0.75 as typical examples to illustrate the behaviors of the generated fGm series.

**Fig 3:**
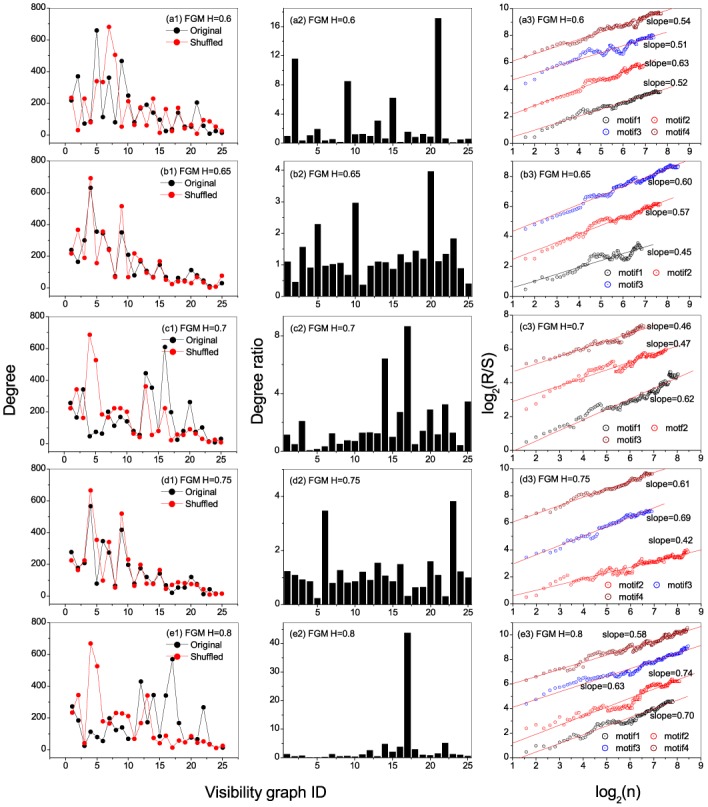
Degree, degree ratio, and persistent behaviors of motifs for fGm series. Segment length is selected to be *s* = 5. (a1)-(e1) show the occurrence degrees of the states in the original and shuffled fGm series with *H* = 0.6, 0.65, 0.7, 0.75 and 0.8, respectively; (a2)-(e2) present the degree ratios for all the states (visibility graphs) in the series with *H* = 0.6, 0.65, 0.7, 0.75, and 0.8, respectively; (a3)-(e3) Relations of *R*/*S* versus *n* obtained from occurring position series of the motifs, from which one can find persistent behaviors of the motifs’ occurring along the series.

Though rather seldom, the self-link is observed in some states, such as the states with id 73(13), 41(20) for *H* = 0.65, and 30(20) for *H* = 0.75. The occurrence of these self-links is weak and thus disappears in the subsequent strong networks. Some states will occur with high frequencies (hubs) in the networks, as represented by the size of nodes in the strong networks (see Fig 2(a2)–2(f2)) (see also the degrees of the nodes in the subplots of Fig 3(a1)–3(e1)). The nodes with Nos 6(4), 7(5), 57(6), and 10(9) are the top four hubs for *H* = 0.65, while the top four hubs for *H* = 0.75 are the nodes with Nos 3(4), 4(9), 10(6) and 52(1). Similarly, over time, certain states will strongly tend to evolve to a specific state as demonstrated by the size of links between any two states. For instance, in the *H* = 0.65, there is strong link between nodes 6(4) and 10(9), and 6(4) and 7(5) (unidirectional), while in the *H* = 0.75 there exists a strong bidirectional link between 3(4) and 4(9). Consequently, these occurrences present us with some degree of immediate predictability, as shown in the strong network (Fig 2(c2)) for *H* = 0.65, the observance of state 6(4) will most likely lead to a behavior similar to 7(5) which in turn may lead to state 57(6).

From the patterns for shuffled networks (see Figs 2(a3)–3(f3)), one can find that the distribution of the increment values alone can also lead to some hubs and strong links between some pairs of states. However, the network patterns for the shuffled series are significantly different with that for the original series. For instance, in the strong network of H = 0.65, the hubs 6(4), 7(5) and 57(6) form a directional transfer loop, while in the shuffling network the node 7(5) is replaced by the state 2(2). The findings from the original series are thus non-trivial, in that the values are series-element-order dependent rather than value distribution dependent.

Fig 3(a2)–3(e2) show the ratios between degrees of local states for the original series versus that in the shuffled ones, from which one can identify the leading motifs. For *H* = 0.65 the leading four motifs are the visibility graphs with ID numbers 41(20), 25(10), 7(5) and 3(23), while that for *H* = 0.75 are the visibility graphs with ID numbers 438(23), 10(6), 30(20) and 21(13). The top four motifs are called herein *motif*1, *motif*2, *motif*3 and *motif*4, respectively.

A natural question is, do the motifs occur randomly along the time series or not? To understand this, we extract the occurring positions of the motifs. The positions for each motif form a time series. We then use the re-scaled range analysis to ascertain the long-term memory behavior in the position series as shown in Fig 3(a3)–3(e3). The identified motifs display the existence of fractal nature. This is exhibited by the existence of power-law behaviors in the relations between *R*/*S* versus *n* of the motifs. For *H* = 0.65, the scaling exponent for *motif*3 (the state 7(5)) is *δ* = 0.60, and for *H* = 0.75 that of *motif*3 (the state 30(20)) is *δ* = 0.69. If we consider only the maximum value *δ*_*m*_ among all the values of scaling exponent for each specified *H* ∈ [0.5, 1], we have *δ*_*m*_ = 0.63, 0.60, 0.62, 0.69, 0.74 corresponding to *H* = 0.6, 0.65, 0.70, 0.75, 0.8. One can find that the maximum value of scaling exponent has a positive cross-correlation with *H*, however, a reliable conclusion requires much more works to be done.

#### Stock market series

**Fig 4:**
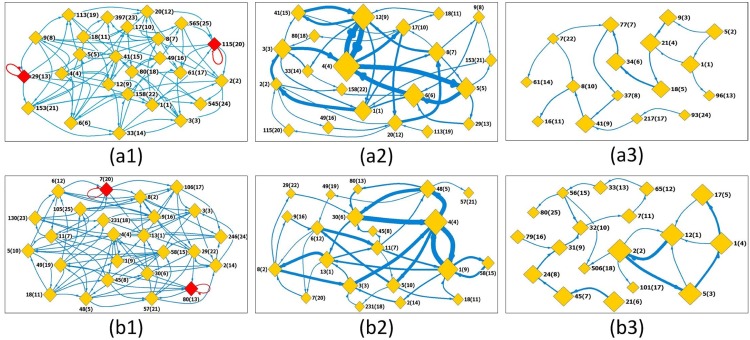
State transfer networks for stock markets. Segment length is selected to be *s* = 5. (a1) and (b1) display the original state transfer networks for S&P500 and Nasdaq respectively. The label *x*(*y*) means the state occurs for the first time at the position *x* along the time series (the *x*’th segment), and its identifier number is *y*; (a2) and (b2) are the strong state transfer networks constructed by filtering out weak links (less than 25) in (a1) and (a2), respectively. While, (a3) and (b3) are the corresponding networks (weak links also are filtered out) constructed from shuffled series. Statistical average is conducted over 1000 realizations. Except in networks shown in (a1) and (b1), the size of a node indicates the occurring degree of the state. The width of an edge is the link’s weight.

Fig 4(a1)–4(a3) and 4(b1)–4(b3) present the state transfer networks, the strong networks, and the shuffled networks for the stock markets S&P500 and Nasdaq respectively. Self-links are observed in states with id 80(13) and 7(20) for the Nasdaq stock index and 29(13) and 115(20) for S&P500 stock index, and disappear in the subsequent strong and shuffled networks. Out of the possible 3737 occurrences, this self reference is observed 12 times for 80(13), 13 times for 7(20) both in Nasdaq; and 7 and 10 times respectively for the two cases of S&P500. Since stock markets are highly dynamic and controlled by various market forces, it is unexpected for a certain state to persist for long hence these weak occurrences are real and acceptable within these networks.

**Fig 5:**
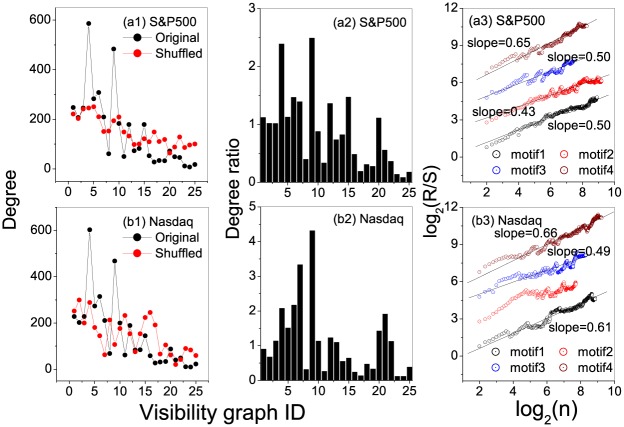
Degree, degree ratio, and persistent behaviors of motifs for the stock markets. Segment length is selected to be *s* = 5. (a1)and (b1) show the occurrence degrees of the states in the original and shuffled S&P500 and Nasdaq index series, respectively. From the occurrence degrees one can easily identify hubs in the original and shuffled series; (a2)-(e2) present the degree ratios for all the states in the S&P500 and Nasdaq index series, respectively. From the ratios one can easily find the the motifs. A state being called a motif means that its degree in the original series is significantly larger than that in the shuffled series; (a3)-(e3) Relations of *R*/*S* versus *n* obtained from occurring position series of the motifs, from which one can find persistent behaviors of the motifs’ occurring along the series.

As can be seen by the size of nodes in the strong networks (see Figs 4, 5(a1) and 5(b1) showing the degrees of the nodes), the nodes with Nos 4(4), 12(9), 5(5), and 6(6) are the top four hubs for S&P500 stock index network, while the top four hubs for Nasdaq stock index are nodes with Nos 4(4), 1(9), 30(6) and 48(5). The significant differences of the link sizes tell us that certain states will strongly tend to evolve to a specific state. For instance, in the S&P500 stock index, there is strong link between nodes 4(4) and 12(9), and 6(6) and 4(4). Whereas in the former the relationship is bidirectional almost in equal measure, the later is only unidirectional. Consequently, the observance of state 6(6) will most likely lead to a behavior similar to 4(4) which in turn may lead to state 12(9). For the two stock markets, we find an identical loop of three hubs linked by strong edges, namely, 4(4)→5(5)→6(6)→4(4) for S&P500 index, and 4(4)→48(5)→30(6)→4(4) for Nasdaq index. Stock markets thus will tend to fluctuate between known behaviors across various times. Actually, there exist some differences in details of the two strong networks, but these differences distribute in the details formed by weak links and non-hubs. The main part of the two strong networks, especially the parts formed by strong links and hubs, are almost identical. What influences a specific behavior and its transitions is an interesting study but not the focus of this work though.

Fig 5(b1) and 5(b2) show the ratios between degrees of local states in the original stock index series versus that in the shuffled ones. One can identify the leading four motifs (*motif*1, *motif*2, *motif*3 and *motif*4), namely, visibility graphs with ID numbers 12(9), 4(4), 41(15) and 6(6) for S&P500 and, visibility graphs with ID numbers 1(9), 11(7), 30(6) and 4(4) for Nasdaq.

From Fig 5(a3) and 5(b3), one can find significant persistence of the motifs’ occurring along the series, as exhibited by the power-laws of *R*/*S* versus *n* in *motif*4 (6(6)) in the S&P500 whose *δ* = 0.65, and also in *motif*1 and *motif*4 (1(9) and 4(4)) for the Nasdaq stock whose *δ* = 0.61 and *δ* = 0.66.

### Window size *s* = 6

**Fig 6:**
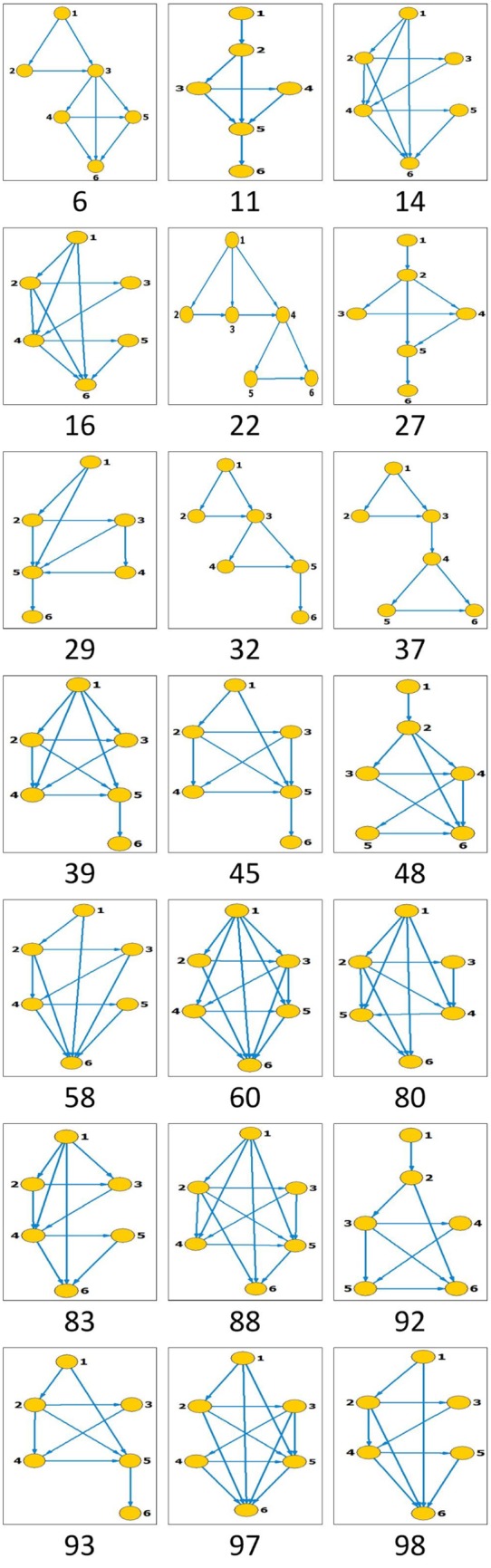
All the states that turn out to be motifs in the fGm series with *H* = 0.6, 0.65, 0.7, 0.75, 0.8 (the total number of occurrence states is 132). Segment length is selected to be *s* = 6.

**Fig 7:**
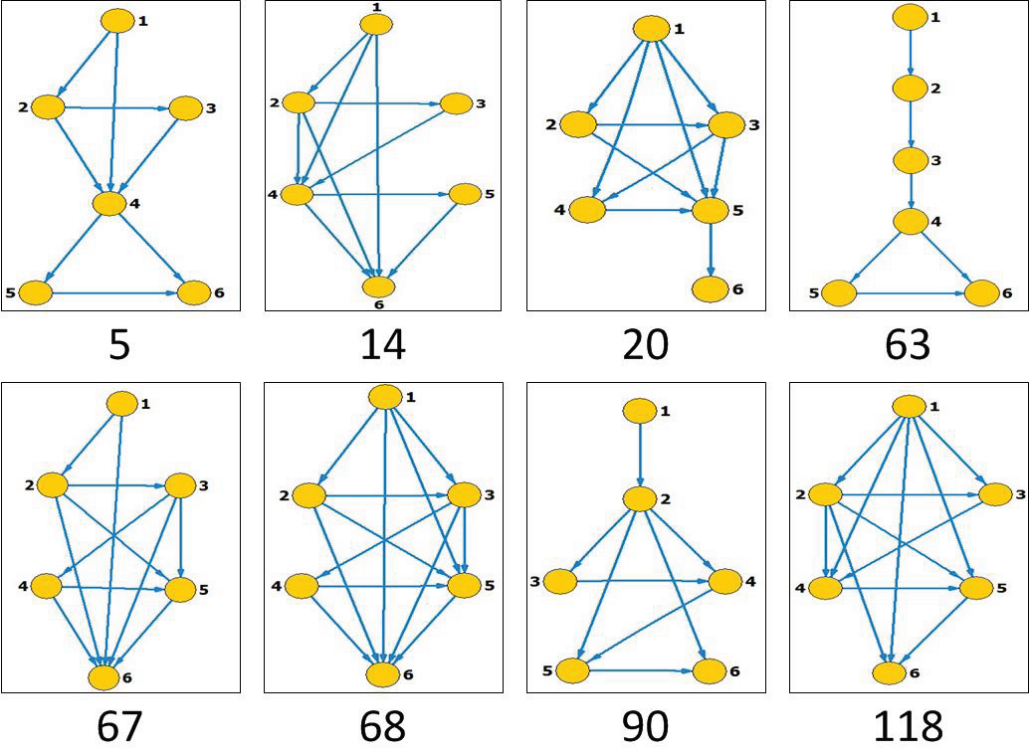
All the states that turn out to be motifs in the stock market index series. Segment length is selected to be *s* = 6.

Like in window size, *s* = 5, for comparison purpose, we re-calculate the results for fGm series with *H* = 0.6, 0.65, 0.7, 0.75 and 0.8, as well those of the empirical data, by using the window size of s = 6. The total number of state patterns that have occurred increases to 132 for *s* = 6 from 25 for *s* = 5. Figs 6 and 7 present only the visibility graphs (with their identifier numbers) that turn out to be motifs in the reference data and the empirical data respectively.

**Fig 8:**
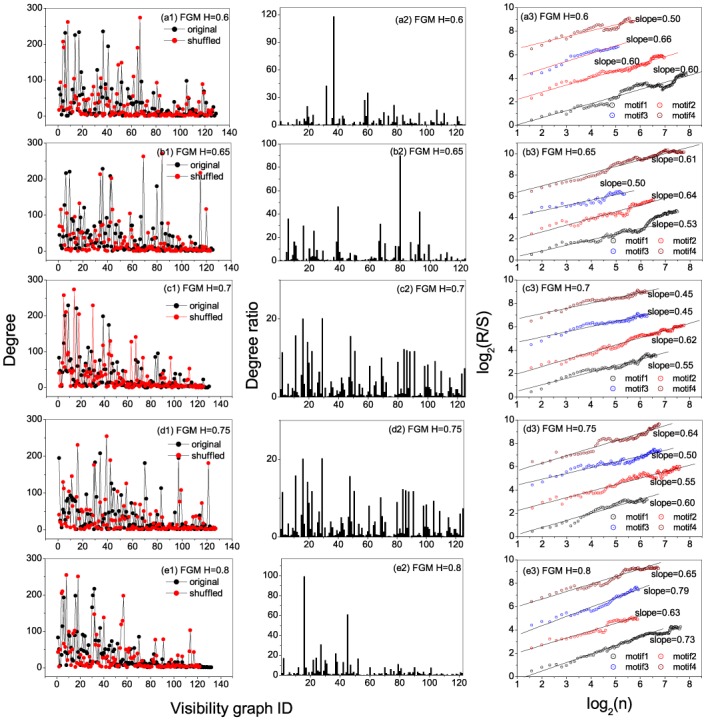
Degree, degree ratio, and persistent behaviors of motifs for fGm series. Segment length is selected to be *s* = 6. (a1)-(e1) show the occurrence degrees of the states in the original and shuffled fGm series with *H* = 0.6, 0.65, 0.7, 0.75 and 0.8, respectively; (a2)-(e2) present the degree ratios for all the states (visibility graphs) in the series with *H* = 0.6, 0.65, 0.7, 0.75, and 0.8, respectively; (a3)-(e3) Relations of *R*/*S* versus *n* obtained from occurring position series of the motifs, from which one can find persistent behaviors of the motifs’ occurring along the series.

**Fig 9:**
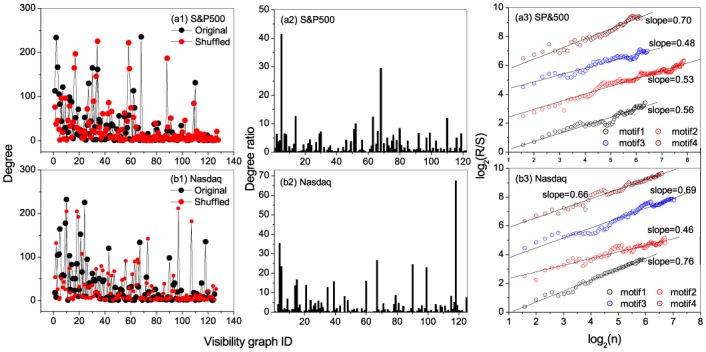
Degree, degree ratio, and persistent behaviors of motifs for the stock markets. Segment length is selected to be *s* = 6. (a1)and (b1) show the occurrence degrees of the states in the original and shuffled S&P500 and Nasdaq index series, respectively. (a2)-(e2) present the degree ratios for all the states in the S&P500 and Nasdaq index series, respectively; (a3)-(e3) Relations of *R*/*S* versus *n* obtained from occurring position series of the motifs, from which one can find persistent behaviors of the motifs’ occurring along the series.

Consequently, the node degrees, degree ratios (original to shuffled) and the persistence of the occurring motifs are presented in Figs 8 and 9 for reference and real data respectively. Referencing the *H* = 0.65 and *H* = 0.75 for the reference data, hubs are observed to be 10(36), 17(9), 6(6) and 57(42); and 32(34), 11(1), 3(97) and 10(30) respectively. The occurring motifs for each can equally be identified. For instance, in *H* = 0.65, the motifs are identified as 80, 39, 93 and 6; and among the four top motifs the nodes 9 and 42 occur along the series according to fractal behaviors with scaling exponents *δ* = 0.64 and 0.61 respectively.

In the empirical data, the top four hubs for S&P500 stock index are identified with the nodes numbered 68, 2, 3 and 30, while the top four motifs are the nodes with nos 5, 68, 14 and 63, among which the forth motif is positioned along the series according to a fractal behavior with *δ* = 0.70. For the Nasdaq stock index, the top four hubs are 10, 24, 9 and 5, the top four motifs are 118, 4, 67 and 90, most of which are positioned along the series according to fractal behaviors (the scaling exponents are 0.76, 0.46, 0.69 and 0.66 respectively).

Comparing the results in both window size, s = 5 and s = 6, the strong networks tend to exhibit (qualitatively) similar structural outlook. However, even so, their evolutionary behaviors are highly varied as shown by the various occurring motifs which are representative of the non-trivial features in the series. Whereas Nasdaq shows a stronger fractal nature, the S&P500 index motifs averagely indicate weak fractality (see Fig 5(a3) and 5(b3) for s = 5 and Fig 9(a3) and 9(b3) for s = 6). Although the close structural nature may be as a result of their regional proximity affected by the same fiscal and political policies, their evolutionary divergence may be attributed to the operational nature of the two indices. While the S&P500 index comprises of the top 500 different companies in the USA, the Nasdaq index mostly represents companies within the IT industry which will mostly tend to fluctuate in same direction. Besides, the divergence in the S&P 500 composition and its weighting methodology while calculating the index may as well have an effect on the behavior of the stock over time hence the difference between the stocks.

## Conclusion and Discussion

Network based time series analysis has reached fruitful achievements in recent years. By mapping mono/multivariate time series into networks one can investigate the time series from microscopic to macroscopic scales. However, the networks generated by most algorithms in literature are static thus cannot provide a detailed account of the evolutionary information on the system. Though there appear limited efforts in constructing transmission networks from mono/multivariate time series in literature, how to use the network viewpoint to extract evolutionary properties embedded in a series still remains an open problem. The study of only structural behaviors as exhibited in most approaches overlooks detailed evolutionary information as well as the ability to conduct short term prediction. These works thus provide the following contributions,

First, we propose a method to convert a mono-variate time series to a weighted network of networks while preserving the local network behavior by using the visibility graph. By this, one can detect evolutionary behaviors of the system. Starting from the phase-space reconstruction procedure, the series segments with a predefined length are converted to visibility graphs as being descriptions of the states corresponding to the time intervals. Linking successively occurring states leads to a state transfer network of distinguishable states. The hubs, motifs, and nontrivial patterns (strong links, loops, etc.) between the nodes tell us the transfer probabilities which are helpful in immediate prediction. The occurring positions of the motifs show the long-term behaviors that are useful in macroscopic prediction.

Second, the paper presents several interesting findings in both empirical and reference data sets. Various nontrivial patterns (hubs, motifs, and loops, etc.) are identified in the fGm networks as well as in the empirical data by using different window sizes. In both cases of window size, s = 5 and s = 6, the findings indicate that the Nasdaq stock index exhibits a more fractal behavior than its counterpart S&P500. Though the structural behavior tend to be similar, their quantitative features are diverse. While their similarity maybe due to their geographical operation, the evolutionary behavior is diverse perhaps due to their varied index computation methods as well as firms composition. Similarly, similar results are exhibited in the generated fGm series for hurst exponent, *H* > 0.5.

Ultimately, by using visibility graphs as being descriptions of local states, the proposed approach maps a time series to a temporal network (network of graph-lets). Accordingly, the theories and tools in the temporal network can be extended to time series analysis, and by extension financial study. By this, the present works present a method that can be used to study various time series research works whose interest is in understanding the evolutionary behaviors of a dynamical system.

## Supporting Information

S1 Matlab Code Code for the algorithm of constructing state transfer network. (RAR)
